# Detection of caprine paratuberculosis (Johne's disease) in pre- and post-vaccinated herds: morphological diagnosis, lesion grading, and bacterial identification

**DOI:** 10.3389/fvets.2024.1395928

**Published:** 2024-07-31

**Authors:** Elena Plamenova Stefanova, Eva Sierra, Antonio Fernández, Oscar Quesada-Canales, Yania Paz-Sánchez, Ana Colom-Rivero, Antonio Espinosa de los Monteros, Pedro Herráez, Lucas Domínguez, Javier Bezos, Marta Pérez-Sancho, Inmaculada Moreno, María A. Risalde, Marisa Andrada

**Affiliations:** ^1^Division of Animal Histology and Pathology, Veterinary School, Institute of Animal Health and Food Safety (IUSA), University of Las Palmas de Gran Canaria, Arucas, Spain; ^2^Departament of Morphology, Veterinary School, University of Las Palmas de Gran Canaria, Arucas, Spain; ^3^VISAVET Health Surveillance Centre, Complutense University of Madrid, Madrid, Spain; ^4^Departamento de Sanidad Animal, Facultad de Veterinaria, Universidad Complutense de Madrid, Madrid, Spain; ^5^Servicio de Inmunología Microbiana, Centro Nacional de Microbiología, Instituto de Investigación Carlos III, Madrid, Spain; ^6^Departamento de Anatomía y Anatomía Patológica Comparadas y Toxicología, Grupo de Investigación GISAZ, UIC Zoonosis y Enfermedades Emergentes ENZOEM, Universidad de Córdoba, Campus de Rabanales, Edificio Sanidad Animal, Córdoba, Spain; ^7^Centro de Investigación Biomédica en Red Enfermedades Infecciosas, Instituto de Salud Carlos III (CIBERINFEC, ISCIII), Madrid, Spain

**Keywords:** *Mycobacterium avium* subspecies *paratuberculosis*, histopathology, Ziehl-Neelsen, immunohistochemistry, molecular diagnosis, vaccination, goat

## Abstract

Samples from the mesenteric lymph nodes (MS LNs) and ileocecal valves (ICV) of 105 goats, comprising 61 non-vaccinated and 44 vaccinated against *Mycobacterium avium* subspecies *paratuberculosis* (MAP), were collected at slaughter from a farm with a confirmed history of paratuberculosis (PTB). These goats had subclinical infections. PTB-compatible lesions in the MS LNs, ICV lamina propria (LP), and Peyer's patches (PPs) were graded separately. Furthermore, the load of acid-fast bacilli was quantified using Ziehl-Neelsen staining (ZN), MAP antigens by immunohistochemistry (IHC), and MAP DNA by PCR targeting the IS900 sequence. Gross PTB-compatible lesions were found in 39% of the goats, with 31.72% vaccinated (V) and 68.29% non-vaccinated (nV). Histopathological lesions induced MAP were observed in 58% of the animals, with 36.07% vaccinated and 63.93% non-vaccinated. The inclusion of histopathology as a diagnostic tool led to a 28% increase in diagnosed cases in MS LNs and 86.05% in ICV. Grade IV granulomas with central mineralization and necrosis were the most common lesions in MS LNs. In the ICV, mild granulomatous enteritis with multifocal foci of epithelioid macrophages was predominant, occurring more frequently in the PPs than in the LP. Furthermore, statistical differences in the presence of histopathological lesions between vaccinated and non-vaccinated goats were noted in MS LNs, ICV LPs, and ICV PPs. Non-vaccinated animals showed higher positivity rates in ZN, IHC, and PCR tests, underscoring the benefits of anti-MAP vaccination in reducing PTB lesions and bacterial load in target organs. Our findings emphasize the necessity of integrating gross and histopathological assessments with various laboratory techniques for accurate morphological and etiological diagnosis of PTB in both vaccinated and non-vaccinated goats with subclinical disease. However, further studies are required to refine sampling protocols for subclinical PTB in goats to enhance the consistency of diagnostic tools.

## 1 Introduction

Paratuberculosis (PTB), caused by *Mycobacterium avium* subspecies *paratuberculosis* (MAP), is a progressive emaciating disease that causes significant economic losses in both cattle and small ruminant industries worldwide (1–5). Although animals usually get infected at an early age due to the high degree of environmental contamination, the subclinical PTB phase is long, and no clinical signs are observed (1–3, 5, 6). Furthermore, once an animal enters the clinical PTB stage, the signs exhibited are non-specific, including diarrhea and severe weight loss (1–3, 5, 6).

Currently, the European Union counts 11,262 thousand goat heads, with the Spanish population being the second largest, with 2,463 thousand heads registered by the end of 2022 (EUROSTAT). The Canary Islands archipelago has the fourth largest goat population in Spain (MAPA 2022), with 232,060 heads and 1,256 farms (ISTAC 2023) of mainly certified autochthonous endangered breeds (Orden APM/26/2018). The overall annual mortality on the canary goat farms in 2017 was approximately 20%, of which 38% was due to PTB (Jiménez, 2017, unpublished data). Although data about the exact prevalence of goat PTB in the continental part of Spain is limited, it is considered a widespread disease that causes considerable economic losses throughout the country (2, 7).

PTB control strategies on goat farms include testing, culling, and vaccination (8). It is demonstrated that vaccination against MAP may considerably reduce the disease-originated economic losses in industrial farms, as well as the severity of the clinical signs and pathological lesions in target organs, although its use has raised controversy since cross-reaction with tuberculosis (TB) on-field diagnostic tests has been described (6, 9–11). Various whole cells were killed, and live attenuated and inactivated vaccines have been developed to prevent PTB in ruminants (6–8, 11–17).

According to the local legislation in the Canary Islands (Decreto 51/2018 del 23 de abril), caprine vaccination is only permitted after “TB-free” status confirmation and PTB diagnostic confirmation since the region was declared “officially free” of bovine TB in 2017. The official testing guidelines establish the comparative intradermal tuberculin (CIT) test as a mandatory exam.

TB and PTB are important livestock concerns worldwide, and goats are especially sensitive to both diseases, with co-infection being reported (18). Thus, proper herd diagnosis is a crucial part of control and prevention strategies (19). Different laboratory tests are used at the herd level for diagnostic confirmation. Indicative clinical signs, evaluation of the host immune response, and necropsy performance are among the commonly used tools. Microbiological culture is the gold standard for PTB detection, but its accuracy is limited in the early PTB stages (19). Molecular biology techniques such as polymerase chain reaction (PCR) targeting the insertion sequence 900 (IS900) have proven to be one of the most sensitive methods for the detection of MAP DNA in different samples, including affected tissues, feces, milk, and buffy coat, although some authors state that its sensitivity decreases when the bacterial load is low (8, 12, 20–22).

Pathological examination is an important tool for adequate confirmation of a postmortem herd diagnosis. Correct evaluation of gross and histopathological findings in tissue samples from affected goats is of utmost importance for a correct diagnosis (12, 23). Routine necropsies are commonly performed to obtain samples for laboratory confirmation and on-field diagnosis via postmortem examination. Gross lesions originating from MAP presence are usually confined to the intestinal mucosa and the draining lymph nodes (12, 24, 25). The intestinal mucosa can be affected by segmental or diffuse lesions distributed from the duodenum to the rectum, although the sites usually affected are the lower ileum and the upper large intestine. The ileocecal valve (ICV) is important for the correct diagnosis, although the detection of gross lesions in this region is variable. The mesenteric (MS) and ileocecal (IC) lymph nodes (LNs) are diffusely enlarged, edematous, and pale. Lymphangitis, which in mild cases can be the only gross lesion present, can be detected with lymphatic vessels being traceable as thickened cords through the MS LNs, as well as intestinal serosa and mesentery (24).

On the other hand, the main MAP-induced histopathological lesions in goats involve transmural granulomatous enteritis, mesenteric and ileocecal granulomatous lymphadenitis, and lymphangitis (12, 24, 25). Intestinal villi can appear moderately to markedly shortened with the infiltration of epithelioid macrophages and a variable number of Langhans-type multinucleated giant cells. The granulomatous infiltrate can have focal or diffuse distribution along the lamina propria, submucosa, muscular layer, or serosa of the intestine. Well-formed granulomas can form and project into the lumen, and in some cases, they undergo central necrosis. The LN lesions can also vary, ranging from foci of epithelioid macrophages up to the final stage of encapsulated granulomas with central necrosis (12, 24, 25).

Since gross lesions are not always present in caprine PTB, correct histopathological assessment is indispensable for adequate postmortem diagnosis of clinical and subclinical cases (7, 12, 23, 26, 27). Thus, various grading systems have been proposed based on the severity and extension of the lesions, the predominant cell type, and/or the bacterial load (12, 13, 28–31). In the case of bovine and ovine PTB, different gradings have been described in both experimental studies and natural infection (13, 29–31). Nevertheless, in the case of natural caprine PTB, the widely used grading proposed by Corpa et al. is based on both the histopathological findings and Ziehl-Neelsen (ZN) quantification (12). To our knowledge, no grading system has been applied in natural cases of goat PTB to evaluate the histopathological findings and the bacterial load separately.

The aim of the present study is to evaluate and grade the PTB-compatible lesions in subclinical goat cases detected at slaughter. Further emphasis is placed on the effects of the anti-MAP vaccination on the development of lesions and the use of grading to optimize early diagnosis. Furthermore, we assess the diagnostic performance of histochemical, immunohistochemical, and molecular techniques for PTB detection in both vaccinated (V) and non-vaccinated (nV) subclinical caprine cases.

## 2 Materials and methods

### 2.1 Herd history

As part of the authorization process for vaccination against PTB, this study was conducted on an intensive dairy goat farm with a census of 3,090 heads located in the Canary Islands, Spain. The local legislation (Decreto 51/2018 del 23 de abril) requires confirmation of PTB presence in the herd as well as certification of TB-free status to grant a vaccination permit. The details are explained in the following sections.

#### 2.1.1 PTB confirmation

The presence of PTB was confirmed at the herd level, as indicated by clinical signs such as weight loss, poor body condition, decreased daily milk production, and diarrhea. More than 50% of the animals tested positive for avian purified protein derivative (aPPD) via the CIT tests. Additionally, serological assays performed on blood serum using the PARACHEK^®^ 2 Kit (Thermo Fisher Scientific, Massachusetts, USA) detected anti-MAP antibodies in over 20% of the animals. Finally, using PCR, MAP DNA was isolated in 35 of the 40 environmental samples tested, contributing to the verification of a TB-free status.

#### 2.1.2 TB-free status confirmation

The absence of TB in the herd was confirmed as requested by the local authorities. A P22 antigenic complex ELISA (Sabiotec, Ciudad Real, Spain) (32) was carried out to detect antibodies against the *Mycobacterium tuberculosis* complex in blood serum. All the results were negative. Moreover, a total of 40 environmental samples were collected using dry sponges (3 M™ Dry-Sponge; 3 M-España, Madrid). Subsequently, DNA was extracted, and PCR was performed on the environmental samples. None of those tested positive for the *Mycobacterium tuberculosis* complex. Finally, an on-field compared CIT test for detection of the *Mycobacterium tuberculosis* complex was performed as requested by the local legislation. All animals with positive or inconclusive results were sent to slaughter. The absence of TB in those animals was confirmed by histopathology and bacterial culture performed by the laboratory of VISAVET, Health Surveillance Centre, Madrid, Spain.

### 2.2 Animals

A total of 105 goats (61 non-vaccinated and 44 vaccinated) were sampled, including 86 animals with positive/inconclusive results on the CIT test and 19 goats with negative results. All of them were euthanized as part of the TB-free status confirmation protocol. Sampling was conducted in two sessions: the first before vaccination, as mandated by local legislation, and a second session 9 months after vaccination implementation. None of the studied animals exhibited PTB-compatible clinical signs, such as severe emaciation, protrusion of lumbar vertebrae, easily palpable transverse processes, muscle mass loss, or reduction in visceral fat deposits. Thus, all cases were classified as “subclinical”.

### 2.3 CIT test

The CIT test was conducted on the farm as per the methodology outlined in previous studies (33), adhering to the Spanish national legislation, R.D. 2611/1996, the European Regulation, EU 2016/429, and the Commission Delegated Regulation EU 2020/688, Orden del 29 de Abril de 2002 de la Consejería de Agricultura y Ganadería de Castilla y León, and Orden AYG/894/2010. Bovine purified protein derivative (bPPD) (0.1 mL; CZ Vaccines S.A., O Porriño, Pontevedra, Spain) was administered on the left side of the neck and avian PPD (0.1 mL; CZ Vaccines S.A., O Porriño, Pontevedra, Spain) on the right side. Readings were taken 72 h post-injection. A positive TB result was indicated by an increase in skinfold thickness at the bPPD injection site on the left side of the neck exceeding 4 mm more than the reaction at the avian PPD site. An 'inconclusive' result for TB was noted if the increase in skin fold thickness at the bPPD site was 8 mm or more or was equal to or less than the reaction at the avian site. Any other reactions were classified as negative.

### 2.4 Anti-MAP vaccine

In the present study, an anti-MAP Gudair^®^ commercial heat-inactivated vaccine containing 2.5 mg/mL of MAP strain 316 F with mineral oil adjuvant (CZ Vaccines S.A., O Porriño, Pontevedra, Spain) for use in sheep and goats was applied. It was administered once subcutaneously following the manufacturer's instructions and the guidelines of the Spanish Agency for Medicines and Medical Devices (AEMPS), which indicate that in heavily affected herds, all animals, including adult ones, should be vaccinated.

### 2.5 Gross examination

An experienced pathologist conducted a macroscopic evaluation of all tissues sampled at the slaughterhouse. Gross lesions were recorded and described in terms of location, color, size, shape, consistency, and number or percent of involvement of the affected organ (34). Subsequently, a presumed morphological diagnosis was established.

### 2.6 Sample collection and processing for histological examination

Samples were collected immediately after the routine slaughter in an abattoir using standard authorized methods detailed in the Spanish national legislation (R.D. 37/2014). LNs and intestines were sampled for histology according to the recommendations of the national bovine TB eradication program protocol 2021 (35) as follows: MS, IC, retropharyngeal (RPh), prescapular (PE), and mediastinal (MD) LNs and ICV. Fresh tissue samples for molecular biology were frozen at −20°C.

For histopathologic examination, tissue samples were fixed in 10% buffered formalin, embedded in paraffin, processed routinely, sectioned at 4 μm, and stained with hematoxylin/eosin (HE).

### 2.7 Histological evaluation of MAP-induced lesions

PTB-compatible histological lesions were graded separately in the MS LNs and the ICV, applying two grading systems. PTB-compatible granulomatous lymphadenitis, ranging from focal to multifocal accumulation of epithelioid macrophages with or without multinucleated giant cells up to encapsulated, well-formed granuloma with central necrosis and mineralization, was classified into four stages. The guidelines followed were the ones described by Wangoo et al. for grading TB LN granulomas: 0 (no lesions), I (initial), II (solid), III (minimal necrosis), and IV (necrosis and mineralization) (36). The ICV affections were graded in terms of severity (mild, moderate, and marked) and distribution (focal, multifocal, and diffuse) using the grading system proposed by Krüger et al., evaluating the lamina propria (LP) and Peyer's patches (PPs) (23) separately. Severity was considered mild when only small circumscribed granulomatous infiltrates were present with no change of tissue architecture; moderate when granulomatous infiltrates with altered tissue architecture were present; and marked when massive granulomatous infiltrates with partially or completely disrupted tissue architecture were observed. The distribution of the lesions was evaluated as focal when up to three distinct granulomatous infiltrates were observed per section, multifocal when more than three distinct granulomatous infiltrates per section were observed, and diffuse when granulomatous infiltrates were present throughout the whole section.

### 2.8 ZN stain and immunohistochemistry (IHC) for MAP confirmation

Selected tissue samples from MS LNs and ICV were sectioned at 4 μm and stained with ZN to detect acid-fast bacilli (AFB) (37).

For immunohistochemistry, samples from the MS LNs and the ICV were sectioned at 3 μm. No epitope retrieval was needed. Inactivation of the endogenous peroxidase was carried out using a solution of 3% hydrogen peroxide in methanol for 30 min in a humidified chamber. Subsequently, immunohistochemical labeling was conducted with a polyclonal anti-MAP in-house antibody kindly provided by Dr. V. Pérez, University of León, León, Spain, diluted 1:2,000 in an antibody diluent (K8006; Dako, Glostrup, Denmark). Sections of ICV samples from PCR-positive MAP-infected goats with PTB histopathological lesions were used as a positive control. The polymer-based detection system that was used (EnVision^®^System Labeled Polymer-HRP; Dako, Glostrup, Denmark) was applied following the manufacturer's guidelines. A commercial solution of 3,3′diaminobenzidine (DAB) (K3468; Dako, Glostrup, Denmark) was used for immunolabelling, and finally, the sections were counterstained with Harris' hematoxylin and mounted in a hydrophobic medium.

The grading score applied to evaluate the number of mycobacteria per section by both IHC and ZN was the one proposed by Krüger et al. (23). Samples were graded as negative when < 2 labeled bacteria were observed per section; as “single” when mycobacteria were present in < 20% epithelioid cells and/or multinucleated giant cells (MGCs) and single/few bacteria per cell or foci of granular labeling were observed, predominantly < 21 μm in diameter; as “few” when mycobacteria was detected in 20% to 50% epithelioid cells and/or MGCs and on average, 1–10 bacteria were present per cell or foci of granular labeling predominantly >21 μmin diameter; and as “many” when mycobacteria was seen in >50% to 75% epithelioid cells and/or MGCs with an average of >10 bacteria per cell, with up to 50% of cells containing countless bacteria (23).

### 2.9 MAP DNA identification

Frozen tissue samples from the MS LNs and ICV were included in a pool sample for DNA extraction. DNA was isolated using the quick-DNA/RNA Magbead extraction kit (Zymo, Irvine, CA, US) following the manufacturer's protocol, which was carried out using the automated extractor TECAN Freedom EVO 200 (Tecan Australia Pty Ltd.).

Subsequently, real-time PCR targeting the IS900 sequence was performed following the protocol previously described by Espinosa et al. (8). Briefly, a mixture of 0.5 μL of 250 nM of forward (MP10-1, [5′-ATGCGCCACGACTTGCAGCCT-3′]) and reverse (MP11-1, [5′-GGCACGGCTCTTGTTGTAGTCG-3′]) primers, 10 μL of PowerUp™ SYBR™ Green Master Mix (Applied Biosystems™, CA, USA), 2 μL of DNA template, and nuclease-free water caused the final volume to be 20 μL. The amplification involved one cycle of 95°C for 8.5 min, 40 cycles of 95°C for 15 s, 68°C for 30 s, and melt curve analysis from 72°C to 95°C using a MiniOpticon™ Real-Time PCR System (Bio-Rad Laboratories, Irvine, CA, USA).

Samples were considered positive when the dissociation peak (Tm) was 89.1 ± 1.5°C, and the threshold cycles (Ct) were ≤ 37. The real-time PCR was performed in triplicate to exclude the negative pool samples.

The PCR products from positive pool samples were purified using a commercial kit (Real Clean Spin Kit 50 Test-REAL) following the manufacturer's protocol. Subsequently, those were sequenced using Sanger DNA sequencing (Secugen S.L., Madrid, Spain). The BLAST database (www.ncbi.nlm.nih.gov/blast/Blast.cgi/) was used to confirm the amplicon identities.

### 2.10 Statistical analysis

An observational cross-sectional study was carried out. The prevalence of macroscopic and microscopic PTB-compatible lesions and their histopathological grading were calculated separately in the MS LNs, the ICV LP, and the ICV PPs.

Statistical analysis was conducted using IBM SPSS Statistics 27 (IBM Corp., released 2020). IBM SPSS Statistics for Windows, Version 27.0. (Armonk, NY, USA: IBM Corp.). Categorical variables were presented as percentages and either relative or absolute frequencies. The ages of the animals were grouped into the following groups: 0–12 months, 12–24 months, 24–36 months, 36–48 months, and >48 months. The association between two categorical variables was analyzed using the Chi-square test, while Kendall's Tau-b test was employed for two ordinal scale variables. Numerical variables were summarized by their mean, standard deviation (SD), median, and interquartile range (IQR). The Shapiro–Wilk test was used to assess the normality of the sample. For non-normal distributions, the Mann–Whitney U test was applied to compare two independent samples. The agreement between qualitative results from two measurement procedures was evaluated using the kappa coefficient (κ). Results were deemed statistically significant if the *p* < 0.05.

## 3 Results

The ages of the goats ranged from 6 to 101 months, with a median age of 14 months, a standard deviation (SD) of 17.60 months, and an interquartile range (IQR) of 7 months. The distribution of goats across age groups was as follows: 31 out of 105 goats were in the 0–12 month group, 53 in the 12–24 month group, 7 in the 24–36 month group, 3 in the 36–48 month group, and 11 in the over 48 month group. Overall, 80% (84 out of 105) of the goats, including 78.70% (48 out of 61) nV and 81.82% (36 out of 44) V animals, were aged between 0 and 24 months.

### 3.1 CIT test

A total of 47.62% of the goats tested positive for the *Mycobacterium tuberculosis* complex using the CIT test, with 34.29% categorized as “inconclusive” and 18.10% categorized as negative. No statistical difference was demonstrated between the ages of the animals and the CIT test results.

The inconclusive group comprised 34.29% of the sample, with a higher proportion of nV animals (83.33%) compared to vaccinated (V) animals (16.67%), a difference that was statistically significant (*p* = 0.001). In contrast, no significant differences were found between the CIT-negative and CIT-positive animals (p = 0.405). Animals with PTB-compatible lesions were identified across the CIT-positive, inconclusive, and negative groups. The association between the CIT test results and the presence of PTB lesions is further explored in Section 3.4.

### 3.2 Gross PTB-compatible lesions

**Figure 1:**
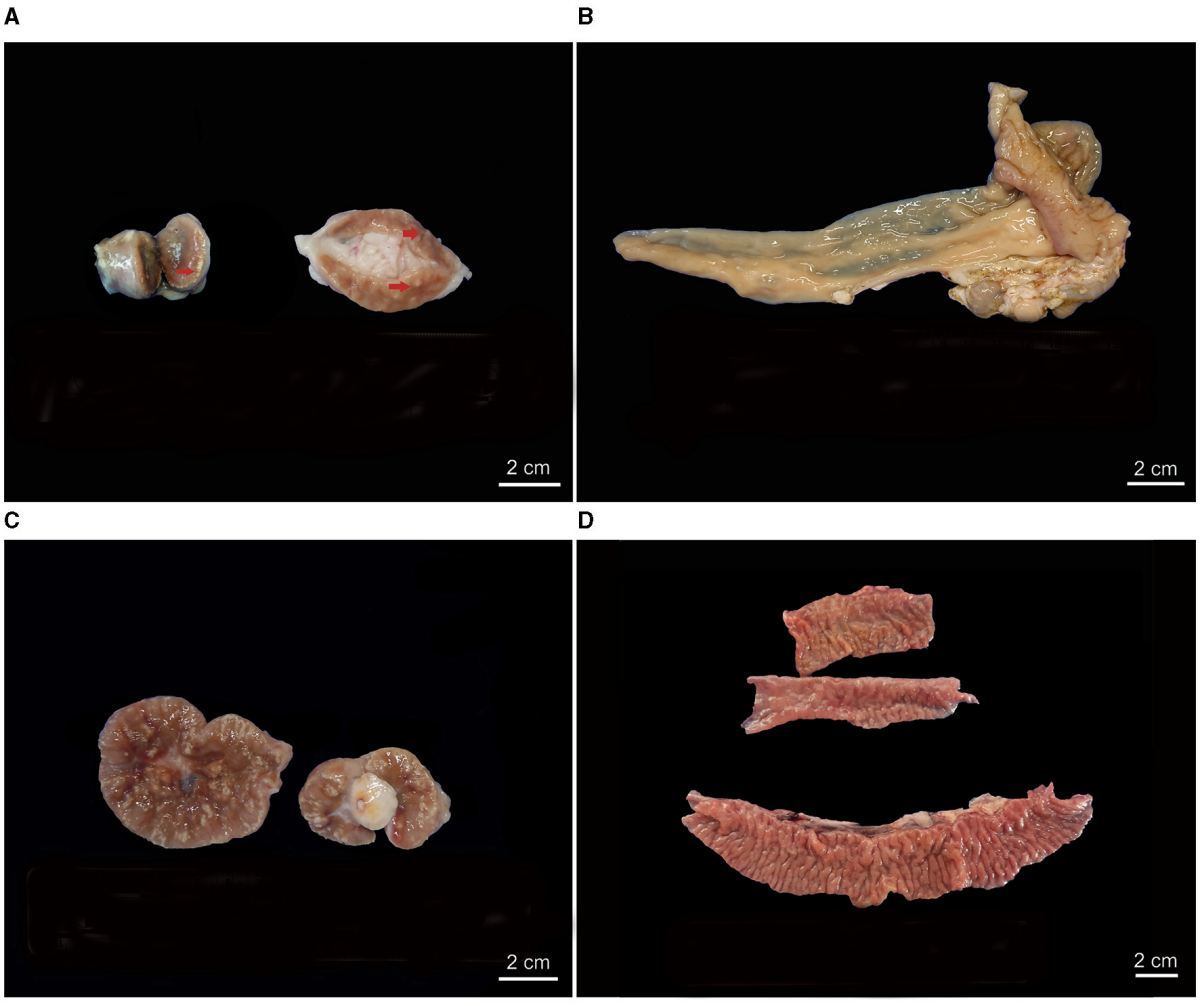
Gross PTB-compatible lesions in vaccinated (V) and non-vaccinated (nV) goats. **(A)** Lymphadenomegaly of mesenteric lymph nodes (MS LNs) of a V animal with multifocal white calcified granulomas on the cut surface (arrow). **(B)** Ileocecal valve (ICV) of a V animal with no gross lesions. **(C)** MS LNs and mesenteric fat of an nV animal exhibiting numerous multifocal to coalescing white calcified granulomas on the cut surface. **(D)** ICV of an nV animal showing diffusely thickened mucosa folded into transverse rugae, characteristic of PTB.

PTB-compatible gross lesions affecting the MS LNs and/or the ICV were found in 39.00% of the animals, of which 68.29% were nV and 31.72% were V (Figure 1). No statistical differences were demonstrated in relation to vaccination status. Gross granulomatous lymphadenitis ranging from small focal granulomas (< 5 mm) with minimal mineralization (Figure 1A) up to well-formed encapsulated calcified multifocal coalescent granulomas (Figure 1C) was observed in 37.14% of animals. On the other hand, the mucosa of the ICV appeared thickened, corrugated, and folded into transverse rugae in 6.67% (Figure 1D) of the goats, in contrast with 93.33% of the animals in which the ICV had no apparent gross lesions (Figure 1B).

### 3.3 Histopathological PTB lesions

**Figure 2:**
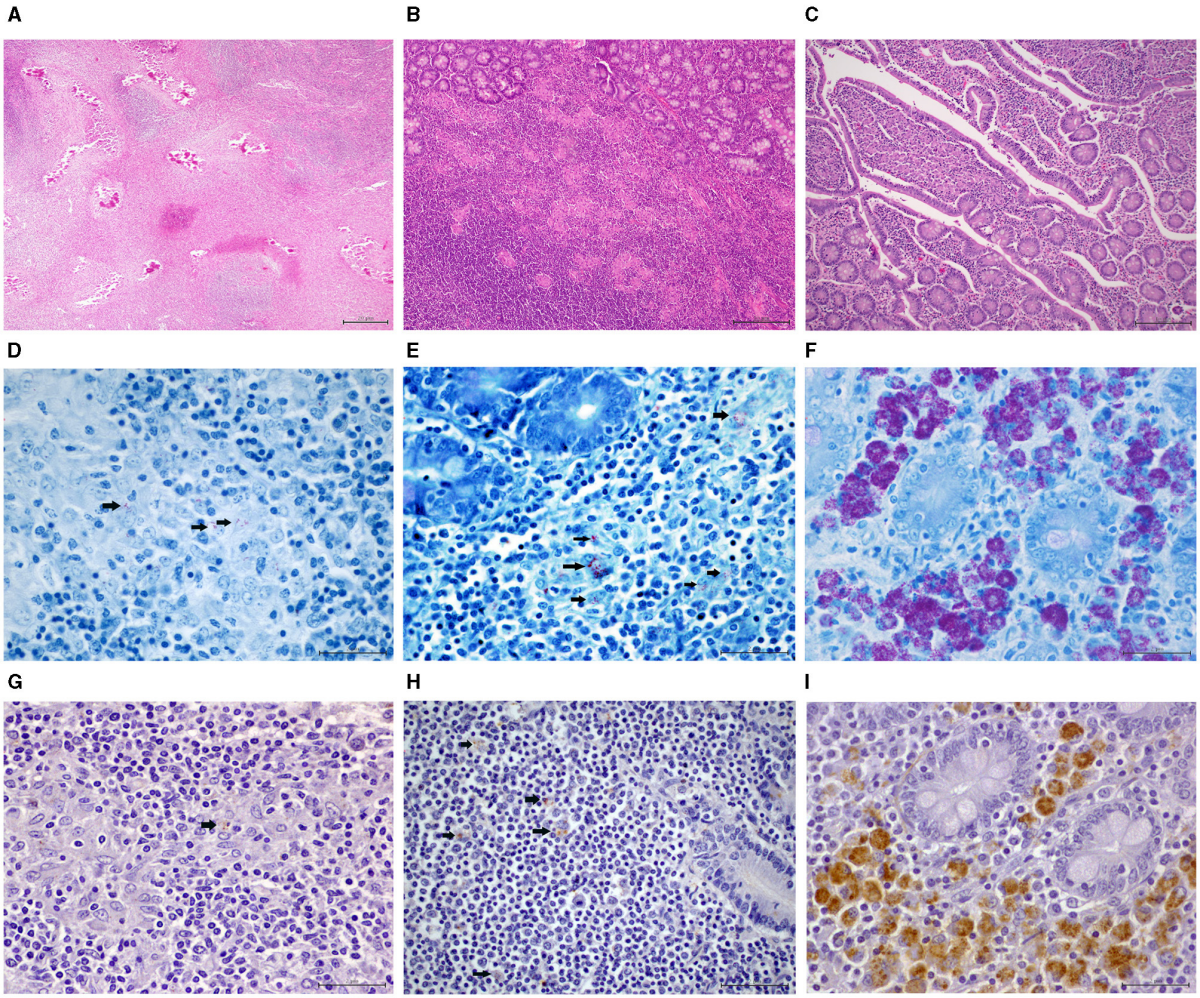
Histopathological PTB-compatible findings in vaccinated (V) and non-vaccinated (nV) goats. **(A)** Mesenteric lymph node (MS LN): grade IV lesions with multifocal severe calcified granulomas. HE 4x **(B)** Peyer's patches (PPs) of the ileocecal valve region (ICV): multifocal mild granulomatous infiltrate of epitheliod macrophages. HE 10x **(C)** Lamina propria (LP) of the ICV: multifocal mild enteritis with epithelioid macrophage aggregates in the tips of the intestinal villi. HE 10x **(D)** MS LN: single acid-fast bacilli (AFB) **(arrows)** present in <20% epithelioid cells and single/few bacteria per cell. Ziehl-Neelsen (ZN) staining, 60x **(E)** ICV, PPs: few AFB detected in 20% to 50% epithelioid cells and multinucleated giant cells (MGCs) with 1 to 10 bacteria present per cell (**arrows**). ZN, 60x **(F)** ICV, LP: AFB is present in >50% to 75% of epithelioid cells and MGCs, with an average of >10 bacteria per cell. ZN, 60x **(G)** MS LN: single mycobacteria (**arrow**) observed in <20% of the epithelioid macrophages. Anti-MAP immunohistochemistry (IHC), 60x **(H)** ICV, PPs: few mycobacteria (**arrows**) detected in 20% to 50% epithelioid cells. IHC, 60x **(I)** ICV, LP: many mycobacteria with strong immunolabeling in >50%−75% epithelioid cells, with approximately 50% of cells containing countless bacteria. IHC, 60x.

**Figure 3:**
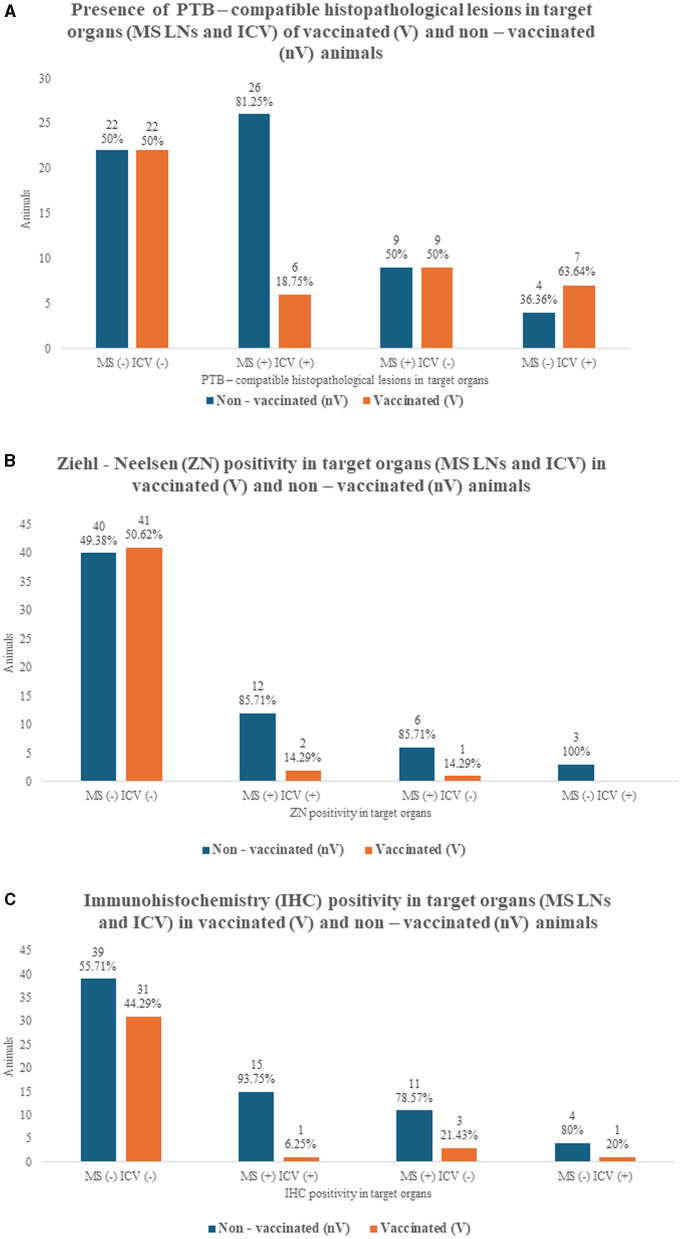
Histopathological PTB-compatible lesions, Ziehl-Neelsen (ZN), and immunohistochemistry (IHC) in target organs of 105 naturally infected goats (vaccinated and non-vaccinated). **(A)** PTB-compatible histopathological findings in mesenteric lymph nodes (MS LNs) and/or ileocecal valve region (ICV) of vaccinated (V) and non-vaccinated (nV) goats. **(B)** ZN positivity in MS LNs and/or ICV of V and nV goats. **(C)** IHC positivity in MS LNs and/or ICV of V goats and nV goats.

The main histopathological findings compatible with PTB are summarized in Table 1, Figures 2, 3. Granulomatous lesions affecting the MS LNs and/or the ICV were detected in 58.10% of the animals, 36.07% were V, and 63.93% were nV (*p* = 0.013). Both MS LNs and the ICV were affected in 52.46% of the animals, with 81.25% nV and 18.75% V (Figure 3A).

**Table 1:** Summary of histopathological PTB-compatible lesions in 105 naturally infected goats (vaccinated and non-vaccinated).

**Lesion site**	**Lesion grading**	**Animals with histopathological; PTB-compatible lesions (%)**	**V**	**nV**	Statistical analysis between groups (*p*-value)^*^
**MS LNs and/or ICV**		61 (58.10%)	22 (36.07%)	39 (63.93%)	**0.013**
**MS LNs**		50 (47.62%)	15 (30%)	35 (70%)	**0.018**
	Grade I	14 (28%)	6 (42.86%)	8 (57.14%)	**0.068**
	Grade II	4 (8%)	-	4 (100%)	
	Grade III	2 (4%)	-	2 (100%)	
	Grade IV	30 (60%)	9 (30%)	21 (70%)	
**ICV LP and/or PPs**		43 (40.95%)	13 (30.23%)	30 (69.77%)	**0.044**
**ICV LP**		23 (21.90%)	5 (21.74%)	18 (78.26%)	**0.027**
Distribution	Focal	2 (8.70%)	2 (100%)	-	**0.004; 0.037** ^**^
	Multifocal	20 (86.96%)	2 (10%)	18 (90%)	
	Diffuse	1 (4.35%)	1 (100%)	-	
Severity	Mild	22 (95.65%)	4 (18.18%)	18 (81.82%)	**0.023; 0.037** ^***^
	Moderate	1 (4.35%)	1 (100%)	-	
	Severe	-	-	-	
**ICV PPs (*****n*** **=** **97)**		39 (40.21%)	11 (28.21%)	28 (71.79%)	**0.028**
Distribution	Focal	8 (20.51%)	6 (75%)	2 (25%)	**0.001**
	Multifocal	31 (79.49%)	5 (16.13%)	26 (83.87%)	
	Diffuse	-	-	-	
Severity	Mild	37 (94.87%)	11 (29.73%)	26 (70.27%)	**0.034**
	Moderate	2 (5.13%)	-	2 (100%)	
	Severe	-	-	-	

nV, non-vaccinated; V, vaccinated; MS LNs, mesenteric lymph nodes; ICV, ileocecal valve region; LP, lamina propria; PPs, Peyer's patches.

^*^The results were considered statistically significant if the p < 0.05.

^**^Significant association between the ages and the severity of PTB-compatible histopathological lesions affecting the ICV LP in the nV group.

^***^Significant association between the ages and the distribution of PTB-compatible histopathological lesions affecting the ICV in the nV group.

The bold text represents statistically significant differences.

The MS LNs presented granulomatous lymphadenitis with microgranuloma and granuloma formation in 47.62% of the goats, and the ICV had granulomatous enteritis with epithelioid macrophages and occasional multinucleated giant cells in LP and/or PPs in 40.95%. Thus, histopathological examination contributed to diagnosing more affected animals in which no gross lesions were described. Specifically, the increase in diagnosed cases was 28% in MS LNs and 86.05% in ICV. Regarding the vaccination status, the V animals appeared less affected histologically in MS LNs (*p* = 0.018), ICV LP (*p* = 0.027), and ICV PPs (*p* = 0.028). In relation to the grading of the PTB-compatible lesions affecting the MS LNs, grade IV encapsulated granulomas with mineralization (Figure 2A) were the most frequent finding (60%), mainly present in the nV group (70%), although the difference was not statistically significant (*p* = 0.068).

In the case of the ICV, the lesions in LP and the PPs were graded as multifocal in 86.96% and 79.49% and mild in 95.65% and 94.87%, respectively (Figures 2B, C). A greater number of nV goats presented lesions in both LP and PPs, with a statistical difference also being demonstrated in terms of grading (Table 1).

### 3.4 Relationship between CIT-test results and PTB-compatible lesions

The main differences were observed in the groups of goats with “inconclusive” CIT test results. More nV goats from this group (66.67%) presented PTB-compatible gross lesions affecting the MS LNs and/or the ICV (*p* = 0.005). Regarding the histopathology results of the same nV animals, 83.33% presented PTB-compatible granulomatous lymphadenitis of the MS LNs and/or granulomatous enteritis affecting the ICV (*p* = 0.008). Thus, 15.67% more nV animals were detected as possibly affected by PTB by adding the use of histopathology as a diagnostic tool. No such difference was demonstrated in the V group (*p* = 0.112), in which only 16.67% of the animals with inconclusive CIT test results had gross PTB-compatible lesions.

In the case of the animals who tested “positive” to the CIT test (47.62%), 52% were V and 48% nV. Gross PTB-compatible lesions were described in 38.46% of the V and in 29.17% of the nV animals. Histopathological PTB-compatible lesions were found in 61.54% of the V and in 45.83% of the nV. No differences were demonstrated regarding the presence of gross or histopathological lesions between the two groups.

In the CIT-negative animals (18.10%), 63.16% of the goats were V and 36.84% nV. Gross PTB-compatible lesions were observed in 16.67% of the V and in 14.29% of the nV. Furthermore, applying histopathology as a diagnostic tool, 41.67% of the V animals and 42.86% of the nV were categorized as goats with PTB-compatible lesions in MS LNs and/or ICV.

### 3.5 MAP identification

#### 3.5.1 ZN staining

The main results of the ZN stain are summarized in Table 2. Red acid-fast bacilli (AFB) were identified in the cytoplasm of epithelioid macrophages, multinucleated giant cells, and/or the mineralized centers of well-formed encapsulated granulomas affecting the MS LNs and/or the ICV in 22.86% of the animals, 12.50% of which were V and 87.50% were nV (p=0.001). A total of 34.43% of the nV goats had PTB-compatible lesions in MS LNs and/or ICV and tested positive on ZN, in contrast with only 6.82% of the V (p=0.001). A total of 63.64% of the ZN-positive animals presented AFB in both MS LNs and ICV, with 14.29% of those V and 85.71% nV (Figure 3B). The ZN positivity was compared between the V and nV animals. Fewer V animals were ZN positive in MS LNs (p=0.004) and in the ICV LP and/or PPs (p=0.006). The AFB load was graded as “single” in 80.95% of the ZN-positive MS LNs, of which only 17.65% were from V animals and 82.35% from nV (p=0.013) (Figure 2D). Regarding the bacterial load in the ICV, 6 of the cases had “single” AFB, 1 had “few,” and 2 had “many” (Figure 2F). The bacterial load in the PPs was graded as “single” in 84.62%, 90.91% of which were from nV animals. Only two goats had “few” (Figure 2E) or “many” AFB, and both were nV.

**Table 2:** Summary of diagnostic test results for the identification of *Mycobacterium avium* subspecies *paratuberculosis* in 105 naturally infected goats (vaccinated and non-vaccinated).

**Lesion site**	**Grading**	**Positive results (%)**	**V**	**nV**	Statistical analysis between groups (p-value)^*^
**Ziehl-Neelsen staining**					
**MS LNs and/or ICV**		24 (22.86%)	3 (12.50%)	21 (87.50%)	**0.001**
**MS LNs**		21 (20.00%)	3 (14.21%)	18 (85.71%)	**0.004**
	Single	17 (80.95%)	3 (17.65%)	14 (82.35%)	**0.013**
	Few	4 (19.05%)	-	4 (100%)	
	Many	-	-	-	
**ICV LP and/or PPs**		17 (16.19%)	2 (11.76%)	15 (88.24%)	**0.006**
**ICV LP**		9 (8.57%)	2 (22.22%)	7 (77.78%)	**-**
	Single	6 (66.67%)	1 (16.67%)	5 (83.33%)	**-**
	Few	1 (11.11%)	-	1 (100%)	
	Many	2 (22.22%)	1 (50%)	1 (50%)	
**ICV PPs (n=98)**		13 (13.27%)	1(7.69%)	12 (92.31%)	**0.028**
	Single	11 (84.62%)	1 (9.09%)	10 (90.91%)	0.076
	Few	1 (7.69%)	-	1 (100%)	
	Many	1 (7.69%)	-	1 (100%)	
**Immunohistochemistry**					
**MS LNs and/or ICV**		35 (33.33%)	5 (14.29%) ^**^	30 (85.71%)^***^	**0.001; 0.018** ^**^ **; 0.001** ^***^
**MS LNs**		30 (28.57%)	4 (13.33%)	26 (86.67%)	**0.001**
	Single	26 (86.67%)	3 (11.54%)	23 (88.46%)	**0.001**
	Few	4 (13.33%)	1 (25%)	3 (75%)	
	Many	-	-	-	
**ICV LP and/or PPs**		21 (20%)	2 (9.52%)	19 (90.48%)	**0.001**
**ICV LP**		15 (14.29%)	2 (13.33%)	13 (86.67%)	**0.015**
	Single	9 (60%)	-	9 (100%)	**0.049**
	Few	4 (26.67%)	1 (25%)	3 (75%)	
	Many	2 (13.33%)	1 (50%)	1 (50%)	
**ICV PPs (n=96)**		17 (17.71%)	2 (11.76%)	15 (88.24%)	**0.006**
	Single	15 (88.24%)	1 (6.67%)	14 (93.33%)	**0.011**
	Few	2 (11.76%)	1 (50%)	1 (50%)	
	Many	-	-	-	
**PCR**					
**MS LNs and/or ICV**		30 (28.57%)	8 (26.67%)	22 (73.33%)	**0.045**

nV, non-vaccinated; V, vaccinated; MS LNs, mesenteric lymph nodes; ICV, ileocecal valve region; LP, lamina propria; PPs, Peyer's patches.

^*^The results were considered statistically significant if the p-value < 0.05.

^**^Fewer V animals with PTB-compatible histopathological lesions tested positive for immunohistochemistry.

^***^More nV animals with PTB-compatible histopathological lesions tested positive for immunohistochemistry.

#### 3.5.2 Immunohistochemistry (IHC)

The main results of the IHC stain are summarized in Table 2. An immunoreaction seen as brown cytoplasmic granules in epithelioid macrophages and/or multinucleated giant cells in lesions affecting the MS LNs and/or the ICV was detected in 33.33% of the studied animals, being only 14.29% V and 85.71% nV (*p* = 0.001). The presence of histopathological PTB lesions and the IHC positivity were compared between the V and nV groups, and statistical differences were demonstrated in animals with lesions in MS LNs and/or ICV (*p* = 0.001) and in those with lesions affecting the ICV LP (*p* = 0.001). Both MS LNs and ICV were IHC positive in 45.71% of the goats, of which 6.25% were V, and 93.75% were nV (Figure 3C).

The differences between the V and nV animals regarding IHC positivity were further analyzed. Fewer V animals were IHC positive in MS LNs (*p* = 0.001), ICV LP, and/or PPs (*p* = 0.001). The MAP load in MS LNs was graded as “single” in 86.67% of the cases, with only 11.54% from V animals and 88.46% from nV (*p* = 0.001) (Figure 2G). Regarding the ICV LP, only two animals presented MAP loads graded as “many” (Figure 2I). In the PPs, 88.24% of the positive samples presented “single” MAP, 93.33% from nV animals and only 6.67% from V (*p* = 0.011), and “few” mycobacteria were detected in only two samples (Figure 2H).

The agreement between the detection capacity of ZN and IHC was moderate (κ = 0.419). Regarding the detection capacity in the MS LNs, the agreement between the two techniques was moderate (κ = 0.513) in contrast with the results from the ICV samples, where the agreement was low (κ = 0.231).

#### 3.5.3 Real-time PCR

A total of 28.57% of the pool samples from mesenteric lymph nodes (MS LNs) and ileocecal valves (ICV) tested positive for the presence of MAP DNA, with 26.63% from V goats, and 73.33% from nV goats (*p* = 0.001) (Table 2). Regarding the presence of PTB-compatible lesions, an association was found only in the nV group, in which only one animal tested positive for PCR but had no MAP-induced lesions in target organs (*p* = 0.001). In the V group, only eight animals tested positive for PCR, and thus, no statistical analysis was conducted due to the small sample size. Nevertheless, five of those goats presented MAP-induced lesions in target organs, while no lesions were found in three.

#### 3.5.4 Relationship between the diagnostic techniques for MAP identification (ZN, IHC, and real-time PCR)

Overall, 52.38% of the animals tested negative by the three techniques for MAP identification; 58.18% of those were V goats, and 41.82% were nV goats (Table 3). 30.91% of the samples that were identified as negative were from animals that presented histopathological lesions compatible with PTB. On the other hand, 11.43% of the animals tested positive by ZN, IHC, and PCR, 16.67% were V goats, and 83.33% were nV goats. All goats in this group presented MAP-induced lesions in target organs. Thus, the three techniques agree in the identification of both positive and negative animals in 63.81% of the cases, 50.75% from V goats, and 49.25% from nV goats. The agreement between PCR and the ZN was low (κ = 0.355), and between PCR and IHC was moderate (κ = 0.444).

**Table 3:** Summary of diagnostic results for MAP identification and presence of histopathological PTB-compatible lesions in 105 naturally infected goats (vaccinated and non-vaccinated).

**MAP identification techniques**	**Histopathological PTB-compatible lesions**	**V**	**nV**	**Total*; N* = 105**
**Agreement between three techniques**				
PCR (-) ZN (-)IHC (-)		32 (58.18%)	23 (41.82%)	55 (52.38%)
	Presence	13 (76.47%)	4 (23.53%)	17 (30.91%)
	Absence	19 (50%)	19 (50%)	38 (69.09%)
PCR (+) ZN (+) IHC (+)		2 (16.67%)	10 (83.33%)	12 (11.43%)
	Presence	2 (16.67%)	10 (83.33%)	12 (100%)
	Absence	-	-	-
Subtotal		34 (50.75%)	33 (49.25%)	67 (63.81%)
	Presence	15 (51.72%)	14 (48.28%)	29 (43.28%)
	Absence	19 (50%)	19 (50%)	38 (56.72%)
**Agreement between two techniques**				
PCR (+) ZN (+) IHC (-)		-	2 (100%)	2 (1.90%)
	Presence	-	2 (100%)	2 (100%)
	Absence	-	-	-
PCR (+) ZN (-) IHC (+)		-	8 (100%)	8 (7.62%)
	Presence	-	7 (100%)	7 (87.50%)
	Absence	-	1 (100%)	1 (12.50%)
PCR (+) ZN (-) IHC (-)		6 (75%)	2 (25%)	8 (7.62%)
	Presence	3 (60%)	2 (40%)	5 (62.50%)
	Absence	3 (100%)	-	3 (37.50%)
PCR (-) ZN (+) IHC (+)		-	5 (100%)	5 (4.76%)
	Presence	-	5 (100%)	5 (100%)
	Absence	-	-	-
PCR (-) ZN (-) IHC (+)		3 (30%)	7 (70%)	10 (9.52%)
	Presence	3 (37.50%)	5 (62.50%)	8 (80%)
	Absence	-	2 (100%)	2 (20%)
PCR (-) ZN (+) IHC (-)		1 (20%)	4 (80%)	5 (4.76%)
	Presence	1 (20%)	4 (80%)	5 (100%)
	Absence	-	-	-
Subtotal		10 (26.32%)	28 (73.68%)	38 (36.19%)
	Presence	7 (21.88%)	25(78.13%)	32 (84.21%)
	Absence	3 (50%)	3 (50%)	6 (15.79%)

MAP, *Mycobacterium avium* subspecies *paratuberculosis*; PTB, paratuberculosis; nV, non-vaccinated; V, vaccinated; PCR, polymerase chain reaction; ZN, Ziehl-Neelsen staining; IHC, immunohistochemistry; (+), positive; (-), negative.

The animals identified as positive by at least one of the three techniques were 36.19%, 26.32% were V goats, and 73.68% were nV goats. Only 6 (three V goats and three nV goats) of those animals did not present PTB-compatible histopathological lesions in MS LNs and/or the ICV. The results are summarized in Table 3.

## 4 Discussion

The direct and indirect economic losses derived from PTB presence in bovine, ovine, and caprine herds have been described worldwide (10, 22, 26, 38, 39). However, diagnostic test sensitivity in the early subclinical stages of the disease is low, and further investigation is needed to address this knowledge gap (6, 22, 40, 41).

CIT tests are widely used in control programs for TB, although their sensitivity is low, and cross-reactions have been described (8, 10, 41, 42). In the present study, a comparative CIT test was used to reduce the risk of non-specific reactions caused by other non-tuberculous mycobacteria (41). Furthermore, although false-positive results originating from the anti-MAP vaccine have been previously reported, no differences were observed between the V and nV CIT-positive animals in our study (8, 10, 41, 42). Nevertheless, statistically, more nV goats with PTB-compatible gross and histopathological lesions presented inconclusive results for aPPD, which confirms the importance of the implementation of the CIT test and the need for subsequent morphological assessment for correct herd diagnosis.

It is worth highlighting that the gross lesions described in this study are not specific to PTB and are not always present in infected animals (5, 6, 11, 12, 24–26). Nevertheless, PTB-compatible gross lesions were mostly detected in nV animals in this study. This might suggest the effect of anti-MAP vaccination in the reduction of PTB lesions and its previously described heterogeneous protective effect in vaccinated herds (8, 11, 14, 17, 43, 44).

In the present study, a considerable percentage of the PTB-compatible lesions could only be detected histologically. Our results highlight the importance of microscopic examination of target organs, as previously stated by other authors (25, 27, 44–46). Furthermore, we used two grading systems to describe the lesions in the MS LNs and the ICV. Goats are particularly susceptible to both PTB and TB, with calcified granulomas being frequently reported, suggesting a limited ability to control the infection (12, 25, 47). Thus, a grading system distinguishing between 4 granuloma stages was applied in this study to classify the severity and the chronic onset of the lesions of MS LNs independently from the amount of mycobacteria present (36).

The MS LNs presented granulomatous lymphadenitis in more nV goats than V goats. Although no such association was demonstrated regarding the grading of the lesions, it is worth pointing out that stage IV granulomas with central necrosis and mineralization were detected in 60% of the affected animals, of which 70% were nV, and only 30% were V. Similar lesions had been frequently reported in caprine PTB in contrast with bovine PTB (12, 24, 45). Our results suggest a possible relationship between the reduction of granulomatous lymphadenitis affecting MS LNs and anti-MAP vaccination. Additionally, the importance of histopathological assessment is confirmed as granulomas can only be grossly detected when they are mineralized (grades III and IV), and those lesions were found to a lesser extent in the V animals. Similar results have been previously reported in both experimental and natural infections, although the histopathological lesions in MS LNs in subclinical cases have not been graded specifically (10, 11, 14, 15, 17, 43).

The grading system applied in this study to evaluate the ICV was chosen as it relies on the morphological description of the lesions detected with HE stain in terms of severity and distribution (23). On the contrary, the widely used score proposed by Corpa et al. is centered on the distribution, the subjectively evaluated intensity, the predominant cell type detected in the lesions, as well as the bacterial load detected by ZN (12). Furthermore, a modification to the initial grading system was implemented, and the LP and the PPs were evaluated separately, as various authors have previously reported differences between the lesions in those two sites in the initial stages of the disease (12, 24, 27). The PPs presented granulomatous lesions in twice as many cases as the LPs. A previous study in naturally infected goats reports that in cases with focal lesions, granulomatous infiltrate is more common in the PPs than in the LP, although not all cases described were subclinical (12). On the other hand, an experimental study focused on the gut-associated lymphoid tissue of PTB cases reported lesions affecting the PPs in 6/7, and it examined 2-year-old animals with no clinical signs (48).

Regarding the vaccination status, statistical differences were demonstrated between the V and the nV animals in relation to the PTB-compatible lesions in both LP and the PPs, with those being classified as mild and multifocal in most cases. Various authors demonstrate the benefit of anti-MAP vaccination on the reduction of losses due to PTB in affected herds (10, 11, 14, 15, 17, 43). In a recent study evaluating the effect of anti-MAP vaccination, only one animal with multifocal lesions affecting both LP and PPs was found, and it was nV, although the authors did not use the same classification as the one applied in our study and conducted the on-field study in a herd with a low prevalence of MAP infection (14). On the contrary, another study conducted in naturally infected goats shows a reduction in the grade of MAP-induced lesions in the target organs of V animals, grading those as mild/multifocal, although not distinguishing between lesion sites (8). In relation to the age of the affected animals, a statistical difference was only demonstrated in the nV group, in which most of the animals with PTB-compatible lesions were between 12 and 24 months of age. Those results must be interpreted with caution as the study was conducted under natural infection conditions, and thus, we have no data about the exact time of infection or the dose of MAP. It is worth highlighting that clinical disease in goat PTB usually appears earlier than in cows (23, 49), although the data about the age of appearance of histopathological lesions is limited. One experimental study found PTB-compatible lesions in goat kids as soon as 3 months post-infection, and those were more severe than in animals evaluated 6 and 12 months post-infection (23). Another experimental study reports lesions in target organs found in subclinical cases 2 years after inoculation (48). In natural infection, clinical and subclinical cases have been reported in animals between 1.5 and 8 years of age (12). The results of our study confirm the chronic onset of the disease in the ICV, although further studies are needed to analyze the effect of vaccination on the age of PTB lesions development.

The identification of MAP in target organs was performed by ZN, IHC, and PCR, and more nV goats than V goats were identified as positive. In the case of the ZN, AFB was detected in the MS LNs and, to a lesser extent, in the ICV, although it is worth mentioning that 13.64% of all ZN-positive animals only presented AFB in the ICV and were nV. The ICV has been described as the only organ histologically affected in the early subclinical stages of goat PTB (11, 12, 24, 27), and our results highlight the importance of its examination for accurate postmortem diagnosis. The ZN negativity in animals with histopathological lesions can be partially explained by the fact that AFB is only stained by ZN when they are intact, as previously reported in both clinical and subclinical cases (28, 50). Furthermore, the differences observed between the V goats and the nV goats in terms of both positivity and amount of AFB might indicate the benefit of vaccination on the reduction of MAP load in target organs. This result can be related to previous studies that reported a decrease in MAP shedding in V herds with both high and low prevalences of MAP infection (10, 11, 17, 43).

The results of the IHC were in line with the ZN ones, detecting statistically more nV animals as positive. The moderate agreement value obtained in our study might be because IHC can detect MAP antigens in ruptured and dead cells, which have been reported in some forms of PTB and other mycobacterial infections due to strong cell-mediated host immunity (3, 12, 50–53). Furthermore, the antibody used in this study is polyclonal, and thus, an unspecific reaction cannot be excluded as previously reported by other authors, which might explain the three cases where IHC positivity was detected in the ICV where no PTB-compatible lesions were seen (12, 50–53). A previous experimental study in sheep inoculated with MAP reported limited detection capacity in focal lesions by both ZN and IHC, although the agreement between the two techniques was not analyzed (3). In the case of goat PTB, some authors report substantial agreement between the two techniques in naturally infected herds, although all of them describe that the cases in which ZN and IHC results disagree presented mild/focal lesions with a small amount of MAP (12, 50, 52).

Finally, real-time PCR agreed with the IHC and ZN staining results in 63.81% of the cases. Regarding the cases where PCR was the only technique that identified the presence of PTB, it is worth mentioning that various studies report its high sensitivity and capacity for the detection of small amounts of DNA, even in cases in which no lesions were detected (3, 5, 8, 54, 55). Nevertheless, the extraction process involves a small tissue sample, in which MAP might be absent in cases with a low bacterial load. Conversely, the histological sections used for both IHC and ZN allow the examination of a larger area, potentially explaining why these methods yielded positive results in 4.76% of the animals that were PCR-negative yet exhibited granulomatous lesions (8, 12, 21, 22, 26, 54). The likelihood of false-negative PCR results due to the absence of the amplified sequence is low in this study, as the IS900 sequence is highly conserved among MAP isolates, and all tissues analyzed were fresh and not formalin-fixed (54, 56). Additionally, despite the general agreement among the three diagnostic techniques, a significant difference was observed between the nV and V groups in terms of MAP positivity, aligning with histopathological findings. These results underscore the beneficial impact of vaccination on reducing bacterial load (10, 11, 17, 43) and demonstrate the importance of using a combination of diagnostic tools to maximize the accuracy of PTB detection in subclinical cases. Nevertheless, further studies are needed to optimize sampling protocols, enhance diagnostic agreement, and thereby improve early detection of MAP infection in goats with PTB.

In conclusion, our study quantifies histopathological lesions in natural cases of goat PTB and highlights the importance of both gross and microscopic examination for a correct postmortem diagnosis. Furthermore, histopathological lesions in both MS LNs and ICV were graded in terms of severity and distribution, demonstrating significant differences between V and nV animals. These results might suggest the potential effect of PTB vaccination on the reduction of histopathological lesions in both MS LNs and ICV in subclinical PTB cases. Moreover, we evaluated the agreement among three laboratory techniques—ZN, IHC, and PCR—for the etiological diagnosis of subclinical PTB cases. Our results demonstrate the crucial role of combining these diagnostic tools to assess subclinical PTB in both vaccinated and non-vaccinated goats accurately.

## Data availability statement

The raw data supporting the conclusions of this article will be made available by the authors, without undue reservation.

## Ethics statement

The requirement of ethical approval was waived by Comité Ético de Experimentación Animal (CEEA-ULPGC), Universidad de Las Palmas de Gran Canaria, Spain for the studies involving animals because according to article 2 of RD 53/2013, non-experimental clinical veterinary practices are excluded from the scope of this Royal Decree, and therefore it does not require approval by an Ethics Committee for Animal Experimentation. The studies were conducted in accordance with the local legislation and institutional requirements. Written informed consent was obtained from the owners for the participation of their animals in this study.

## Author contributions

EPS: Data curation, Formal analysis, Investigation, Methodology, Project administration, Validation, Writing – original draft, Writing – review & editing. ES: Investigation, Methodology, Supervision, Validation, Writing – review & editing. AF: Funding acquisition, Resources, Supervision, Writing – review & editing. OQ-C: Investigation, Methodology, Supervision, Writing – original draft, Conceptualization, Funding acquisition. YP-S: Data curation, Investigation, Methodology, Project administration, Supervision, Writing – original draft. AC-R: Methodology, Writing – review & editing. AE: Conceptualization, Funding acquisition, Supervision, Writing – review & editing. PH: Conceptualization, Methodology, Supervision, Writing – review & editing. LD: Conceptualization, Formal analysis, Investigation, Supervision, Writing – review & editing. JB: Conceptualization, Formal analysis, Investigation, Supervision, Writing – review & editing. MP-S: Formal analysis, Investigation, Supervision, Writing – review & editing. IM: Formal analysis, Investigation, Supervision, Writing – review & editing. MR: Formal analysis, Investigation, Supervision, Writing – review & editing. MA: Data curation, Formal analysis, Funding acquisition, Investigation, Methodology, Project administration, Supervision, Validation, Writing – original draft, Writing – review & editing.
